# Chemically inducible antisense oligonucleotides for cell-specific gene silencing

**DOI:** 10.1039/d5cb00186b

**Published:** 2025-10-09

**Authors:** Zhen Xun, Yang Hai, Li-Juan Tang, Jian-Hui Jiang, Zhenkun Wu

**Affiliations:** a State Key Laboratory of Chemo and Biosensing, College of Chemistry and Chemical Engineering, Hunan University Changsha 410082 China tomwu@hnu.edu.cn

## Abstract

Cell-specific control of the function of antisense oligonucleotides (ASOs) is highly desirable for precise gene therapy while minimizing adverse effects in normal cells. Herein, we report a novel class of chemically inducible ASOs (iASOs) that achieve tumor-cell-selective gene silencing through hydrogen peroxide (H_2_O_2_)-triggered activation. Through post-synthetic incorporation of phenylboronic acid (BO) caging groups at the backbone positions, we developed iASOs that remain functionally inactive until the H_2_O_2_-triggered removal of the BO groups caused activation. Using EGFP as a reporter system, we demonstrated that the optimal BO-modified iASO exhibited slight gene silencing activity in normal cells but achieved >80% knockdown of the target mRNA in tumor cells. The BO-modified iASO was further applied to target the endogenous Bcl_2_ gene, demonstrating its ability for controlling gene silencing and inducing cell death. This study establishes a simple and effective platform for conditional gene regulation and the development of cell-specific ASO therapeutics.

## Abstract 2

Chemically inducible antisense oligonucleotides (iASOs) have been developed by site-specific incorporation of phenylboronic acid (BO) groups at backbone positions, thereby enabling precise and controllable gene silencing in live cells.

## Graphical abstract

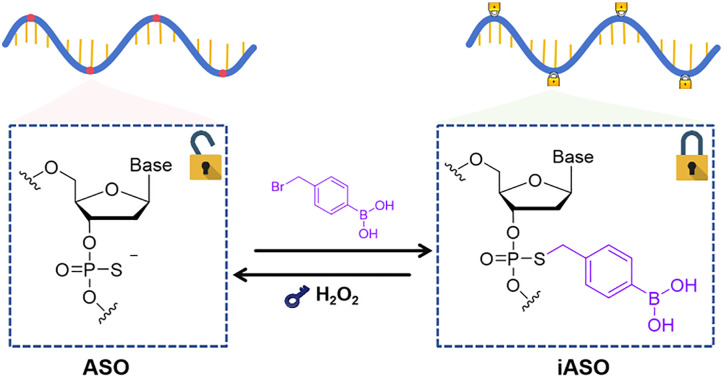

## Introduction

Antisense oligonucleotides (ASOs) have emerged as highly promising therapeutic agents for regulating gene expression.^1–7^ ASOs are single-stranded synthetic nucleic acids that specifically hybridize with their target mRNA through Watson–Crick base pairing, thereby modulating gene expression through several mechanisms, including ribonuclease H-mediated mRNA degradation, RNA splicing modulation and the steric inhibition of mRNA translation.^8–12^ Owing to their high programmability, ASOs offer exceptional targeting versatility that allow for the rational design of almost any mRNA of interest. Currently, several ASO drugs, such as Inotersen,^13^ Volanesorsen,^14,15^ and Nusinersen,^16,17^ have been approved, and an increasing number of drug candidates targeting different diseases have also entered clinical trials.^18,19^ Despite their promise, a critical pharmacological limitation of ASOs is their constitutive activity; that is, upon cellular uptake, therapeutic ASOs induce rapid and sustained gene silencing without precise control of their functions. This “always-on” characteristic raises the risk of off-target effects and systemic toxicity, particularly in non-target tissues where prolonged ASO activity may result in adverse effects.

To address this issue, several strategies that enable the precise control of ASO function have been developed. For example, aptamer-mediated conformational switches have been designed that allow for the regulation of gene expression through exposure of the antisense sequence, triggered by ligand binding.^20,21^ An alternative approach is constructing chemically caged ASOs through the site-specific incorporation of stimuli-responsive protecting groups at the conserved positions of ASOs. These engineered ASOs would remain inactive under physiological conditions until specific stimuli trigger decaging, thereby activating their functions. Photoactivated ASOs have been developed by incorporating photocleavable groups into the bases and phosphodiester linkages or by constructing cyclized ASOs that inhibit binding of mRNA to the target.^22–26^ Although these strategies enable the spatiotemporal control of the functions of ASO for conditional gene silencing in cells, their biological applications are largely limited by the requirement for short-wavelength irradiation, which causes poor tissue penetration and potential phototoxicity.^27,28^ Alternative strategies involving small-molecule-activated ASOs have demonstrated efficient deprotection *via* the Staudinger reduction, enabling controlled gene silencing in live cells and zebrafish models.^29,30^ However, the dependence on potentially toxic phosphine-based triggers raises significant safety concerns for clinical applications.^31^ More recently, endogenous stimulus-responsive ASO systems, utilizing either chemically modified nucleosides or circular ASOs with cleavable linkers, have been developed for cell-specific gene regulation.^32–35^ For example, Obika *et al.* designed a series of boronated nucleosides for constructing a H_2_O_2_-activated ASO *via* a solid-phase synthesis.^32^ Despite their biological relevance, these approaches often involve complex syntheses that may hinder their practical implementation. Therefore, a simple, robust, and effective strategy for achieving cell-selective control of ASO functions remains highly desirable.

**Scheme 1:**
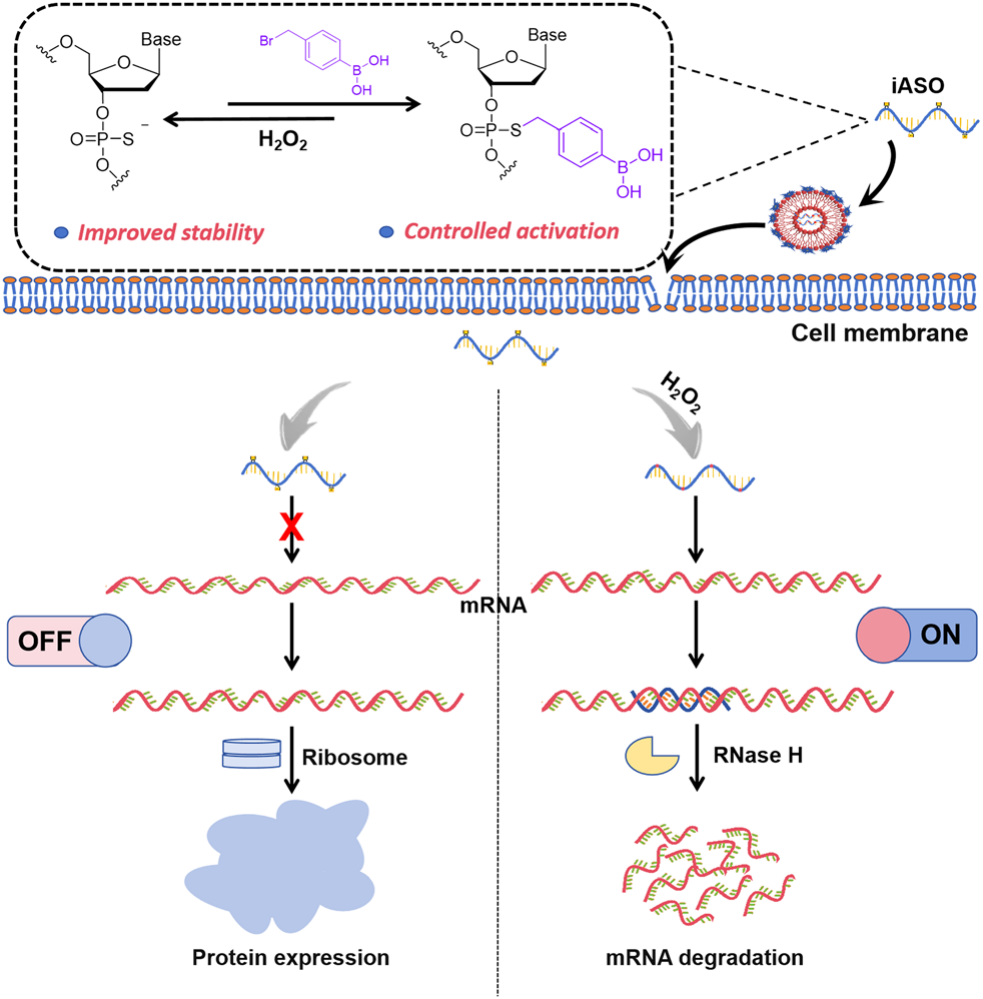
Schematic of the H_2_O_2_-triggered activation of chemically inducible ASO for controlled gene silencing.

In this study, we present a chemically inducible ASO (iASO) design that relies on the site-specific incorporation of caging groups at the backbone positions of ASOs, allowing for the steric hindrance of mRNA hybridization and utilization of endogenous biomolecule-triggered decaging to restore functionality (Scheme 1). As a proof of concept, we selected H_2_O_2_ as a model stimulus. H_2_O_2_ is a metabolic by-product with elevated levels in tumor cells,^36^ making it a promising trigger for regulating the function of various molecules, such as small molecules, proteins and nucleic acids.^37–45^ We designed a series of phenylboronic acid (BO)-modified ASOs *via* effective phosphorothioate (PS)-bromide chemistry,^46^ and subsequently identified an iASO with a relatively low basal activity leakage. The H_2_O_2_-triggered oxidative hydrolysis of BO enabled the effective removal of the caging groups,^47^ thereby activating the functioning of ASO (Fig. S1). Our results demonstrated that iASOs exhibited remarkably reduced hybridization capacity and enhanced structural stability in complex biological environments. Upon H_2_O_2_-triggered activation, these iASOs enabled the conditional downregulation of the targeted gene expression in tumor cells. Using enhanced green fluorescent protein (EGFP) as an optical reporter, minimal off-target effects were observed in normal cells. Furthermore, we applied this strategy to an inducible ASO that targeted Bcl_2_, an endogenous, anti-apoptotic gene. The H_2_O_2_-triggered activation induced remarkable Bcl_2_ silencing and subsequent tumor cell death, underscoring the therapeutic potential of this approach. Collectively, our findings establish a simple and effective platform for controllable gene silencing with applications in precision tumor therapy.

## Results and discussion

### Design of H_2_O_2_-inducible A_GFP_@4BO

To demonstrate the feasibility of constructing iASOs through the site-specific incorporation of caging groups at the backbone positions, we selected EGFP as an optical reporter for evaluating the gene-silencing efficiency. Eight ASO candidates targeting EGFP were initially assessed to identify the most effective sequence for gene regulation. HEK293T cells were co-transfected with EGFP plasmids and each ASO candidate individually, followed by analysis using fluorescence confocal microscopy and flow cytometry. The results indicated that the ASO candidates exhibited varying levels of gene silencing efficiency, with A_GFP-8_ achieving the highest inhibition rate, as determined by the proportion of GFP-positive cells (Fig. S2). Therefore, A_GFP-8_ was chosen for subsequent experiments.

**Fig. 1:**
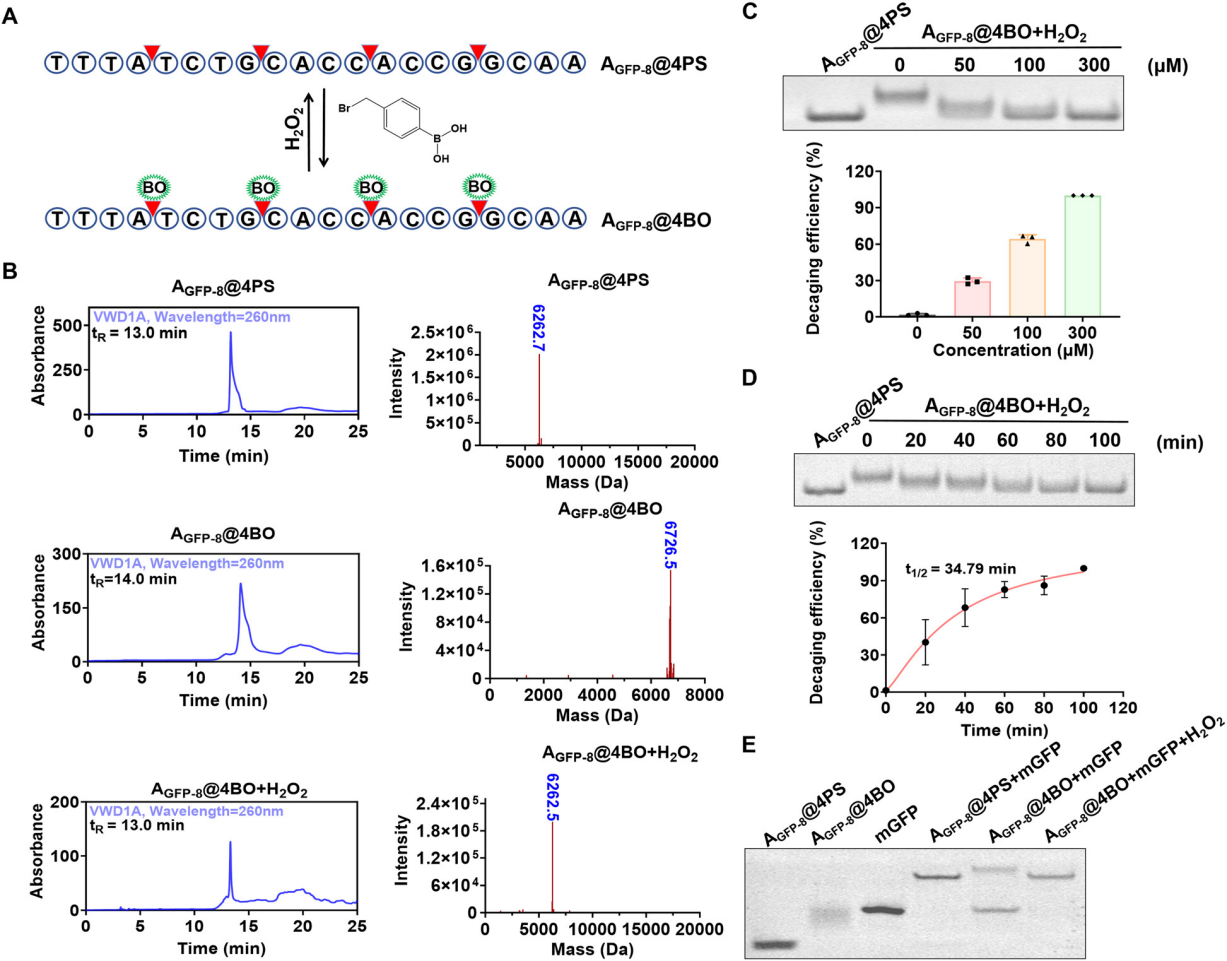
Characterization of the BO-modified iASO. (A) Schematic of the H_2_O_2_-triggered activation of iASO. (B) HPLC (left column) and MS (right column) characterization of A_GFP-8_@4BO and the H_2_O_2_-triggered decaging products (300 μM H_2_O_2_ and 100 min). The observed molecular weight of A_GFP-8_@4BO was smaller than the theoretical value, likely due to the formation of the dehydrated species during ESI-MS analysis. (C) Representative gel image of various concentrations used for the H_2_O_2_-triggered decaging of A_GFP-8_@4BO (top panel). Calculated dose-dependent decaging efficiency (bottom panel). (D) Representative gel image of the H_2_O_2_-triggered decaging of A_GFP-8_@4BO at different times (top panel). The calculated time-dependent decaging efficiency (bottom panel). (E) Representative gel image of the H_2_O_2_-triggered activation of iASO for target mRNA hybridization. Error bars represent standard deviation from the three parallel experiments. The decaging reactions were performed in 1× Tris-NaCl buffer: 10 mM Tris-HCl, 140 mM NaCl, and pH 7.4.

To engineer a H_2_O_2_-activatable iASO, we initially synthesized a phosphorothioate (PS)-modified A_GFP-8_ containing four evenly spaced PS modifications (designated as A_GFP-8_@4PS). The PS modifications not only enhanced the stability of the oligonucleotide, but they also provided reactive sites for the subsequent conjugation of the BO group *via* bromide-PS chemistry, resulting in BO-modified ASOs (designated as A_GFP-8_@4BO, Fig. 1A). The synthesis of A_GFP-8_@4BO was characterized using high performance liquid chromatography (HPLC) and mass spectrometry (MS) analyses. The results show that A_GFP-8_@4BO exhibited a longer retention time (∼14 min) compared to A_GFP-8_@4PS (∼13 min), consistent with the increased hydrophobicity conferred by the BO groups. Notably, H_2_O_2_ treatment restored the retention time to ∼13 min, indicating that the H_2_O_2_-triggered removal of the BO groups (Fig. 1B, left column, Fig. S1). The MS spectra confirmed these observations (Fig. 1B, right column). Collectively, these results demonstrate the feasibility of constructing chemically inducible ASOs.

The dose- and time-dependent decaging efficiencies were evaluated. The incubation of A_GFP-8_@4BO with varying concentrations of H_2_O_2_ for 100 min resulted in a progressively increased decaging efficiency, with 300 μM H_2_O_2_ achieving ∼99% decaging efficiency (Fig. 1C). Similarly, incubation with 300 μM μH_2_O_2_ over different time periods revealed a time-dependent decaging process with a *t*_1/2_ of 34.79 min (Fig. 1D). These results confirm that the H_2_O_2_-triggered decaging reaction occurred in both dose- and time-dependent manners. The specificity of the H_2_O_2_-triggered decaging was also investigated. The result show that upon H_2_O_2_ treatment, A_GFP-8_@4BO exhibited a remarkably increased bond migration rate compared to A_GFP-8_@4BO, whereas the other oxidizing agents induced minimal effects (Fig. S3). This result demonstrates the good selectivity of the BO-modified ASO for H_2_O_2_, consistent with the previous reports that the BO group serves as an H_2_O_2_-responsive motif.^48^

We further investigated the ability of A_GFP-8_@4BO for modulating hybridization with the target mRNAs (Fig. 1E). The results demonstrated that A_GFP-8_@4BO significantly inhibited hybridization activity toward mEGFP (lane 5), whereas H_2_O_2_ treatment fully restored this activity (lane 6). The DNA melting analysis revealed that A_GFP-8_@4BO exhibited a ∼10 °C drop in the melting temperature compared to A_GFP-8_@4PS, suggesting that the incorporation of the BO groups into ASO significantly blocked its hybridization capacity (Fig. S4). Furthermore, the H_2_O_2_-activated A_GFP-8_@4BO showed a recovered melting temperature comparable to that of A_GFP-8_@4PS. These findings together underscore the potential of A_GFP-8_@4BO for achieving controllable gene silencing. Moreover, the structural stability of A_GFP-8_@4BO was further enhanced due to increased steric hindrance conferred by the chemical modifications. As shown in Fig. S5, ∼70% of A_GFP-8_@4BO remained intact after a 4-h incubation in 10% fetal bovine serum (FBS) solution, which was higher than that of A_GFP-8_@4PS and A_GFP-8_.

Encouraged by the results of H_2_O_2_-triggered activation of ASO functions, we further assessed the potential of A_GFP-8_@4BO for controlled gene silencing in live cells, using EGFP as an optical reporter. Prior to conducting experiments, the cytotoxicity of H_2_O_2_ was evaluated in HEK293T cells to determine the maximum concentration (maintaining ∼90% cell viability) that could be used for subsequent activation experiments (Fig. S6A). The results indicate that A_GFP-8_@4PS caused a relatively higher reduction in EGFP expression in HEK293T cells compared to A_GFP-8_ (Fig. S7), primarily due to the enhanced structural stability provided by the PS modifications. Furthermore, only A_GFP-8_@4BO had a slight impact on EGFP expression, whereas sequential treatment with A_GFP-8_@4BO and H_2_O_2_ led to a significant decrease in EGFP levels in HEK293T cells (Fig. S7). Notably, only cells transfected with EGFP plasmids exhibited negligible changes in their fluorescence under identical H_2_O_2_ treatment conditions (Fig. S6B). These results confirm the H_2_O_2_-triggered activation of A_GFP-8_@4BO for controllable gene silencing in living cells.

### Live cell characterization of H_2_O_2_ inducible A_GFP_@4BO

**Fig. 2:**
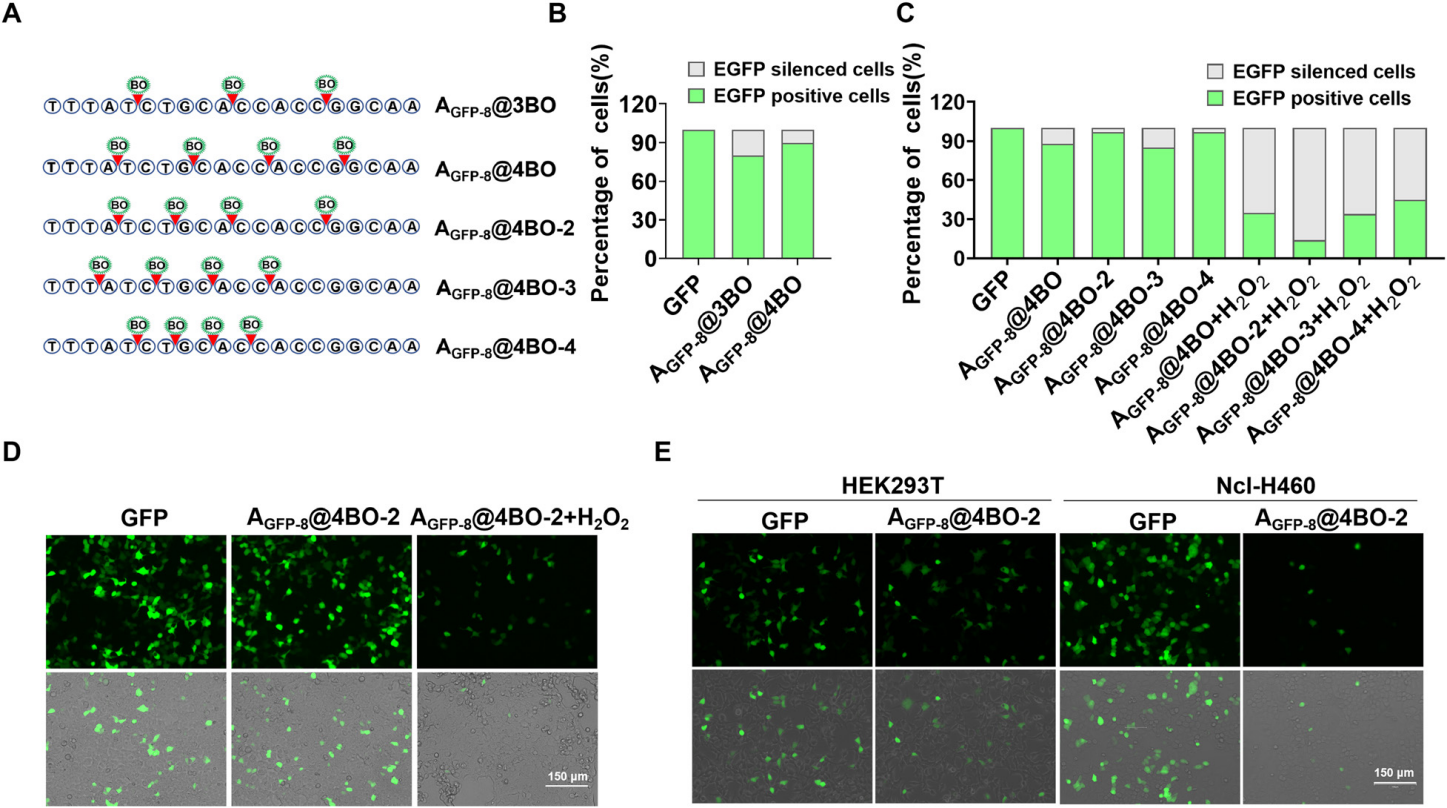
H_2_O_2_-triggered activation of A_GFP-8_@4BO for cell-specific gene silencing of exogenous EGFP mRNA. (A) Schematic of the BO modifications on ASOs. Gene silencing efficiency of the iASO candidates with different modification numbers (B) or different modification sites (C), as measured by flow cytometric analysis of GFP expression in HEK293T cells. (D) Confocal imaging of HEK293T cells transfected with A_GFP-8_@4BO-2, followed by treatment with or without H_2_O_2_. (E) Confocal imaging of HEK293T and Ncl-H460 cells transfected with A_GFP-8_@4BO-2.

We then optimized the number and sites of chemical modifications to identify the optimal iASO. The results indicate that A_GFP-8_ containing three evenly spaced BO modifications (designated as A_GFP-8_@3BO as shown in Fig. 2A, S8A and S8B) exhibited noticeable activity leakage in HEK293T cells, whereas A_GFP-8_@4BO demonstrated much lower gene silencing activity (Fig. 2B and Fig. S8C), suggesting that at least four BO modifications were required to effectively suppress its function. The influence of modification sites on the gene-silencing performance was also examined. The results revealed that the A_GFP-8_@4BO variants with four BO modifications at different backbone positions displayed variable inhibition and activation efficiencies (Fig. 2A and Fig. S9). Among these variants, A_GFP-8_@4BO-2 showed the lowest basal activity leakage prior to H_2_O_2_-triggered decaging, and subsequent H_2_O_2_-triggered decaging enabled the efficient activation of gene silencing (Fig. 2C, D and Fig. S10). Collectively, these results suggest that A_GFP-8_@4BO-2 functioned as an optimal iASO for controlled gene regulation in living cells.

It has been shown that the overproduction of H_2_O_2_ is closely associated with various diseases including cancer, making it a widely utilized stimulus for activating prodrugs in tumor therapy. We subsequently evaluated the ability of A_GFP-8_@4BO-2 for achieving tumor cell-specific gene regulation. A human lung carcinoma cell line, Ncl-H460, and a normal human embryonic kidney cell line, HEK293T, were selected as model cell lines for comparison. Compared to HEK293T cells co-transfected with EGFP plasmids and A_GFP-8_@4BO-2, which showed only a modest reduction in EGFP expression (∼20% gene silencing efficiency), cancerous Ncl-H460 cells co- transfected with the same constructs exhibited a substantial decrease in EGFP fluorescence, corresponding to ∼80% gene silencing efficiency (Fig. 2E and Fig. S11A–C). This result was consistent with the intracellular H_2_O_2_ levels in these two cell lines, as quantified using a commercially available H_2_O_2_ assay kit (Fig. S11D). Collectively, these results demonstrate the H_2_O_2_-triggered activation of iASO for tumor cell-specific gene silencing.

### Live cell gene silencing of Bcl_2_ using H_2_O_2_ inducible A_Bcl_2__@BO

Having demonstrated the efficient gene silencing of the exogenous mRNA, we proceeded to design another iASO that targeted the endogenous mRNA, Bcl_2_, a key regulator of cell apoptosis. Previous studies have established that Bcl_2_ plays a critical role in promoting cell survival in most mammalian cells and contributes to chemoresistance in cancer.^49^ The gene regulation capability of the previously reported Bcl_2_-targeting ASO, G3139^50^ was first validated (Fig. S12). The BO-modified A_Bcl_2__ was then synthesized according to the developed method (designated as A_Bcl_2__@BO, Fig. S13A) and characterized by HPLC and MS analyses (Fig. S13C). Additionally, a negative control A_Bcl_2__ modified with phenylacetic acid groups, which cannot respond to H_2_O_2_ stimulation, was constructed (designated as A_Bcl_2__@CC, Fig. S13B and S13C). Consistent with previous observations, A_Bcl_2__@BO exhibited significantly reduced hybridization activity (Fig. S13D), which was restored upon H_2_O_2_-triggered activation. In contrast, the hybridization capacity of A_Bcl_2__@CC remained constitutively suppressed (Fig. S10E).

**Fig. 3:**
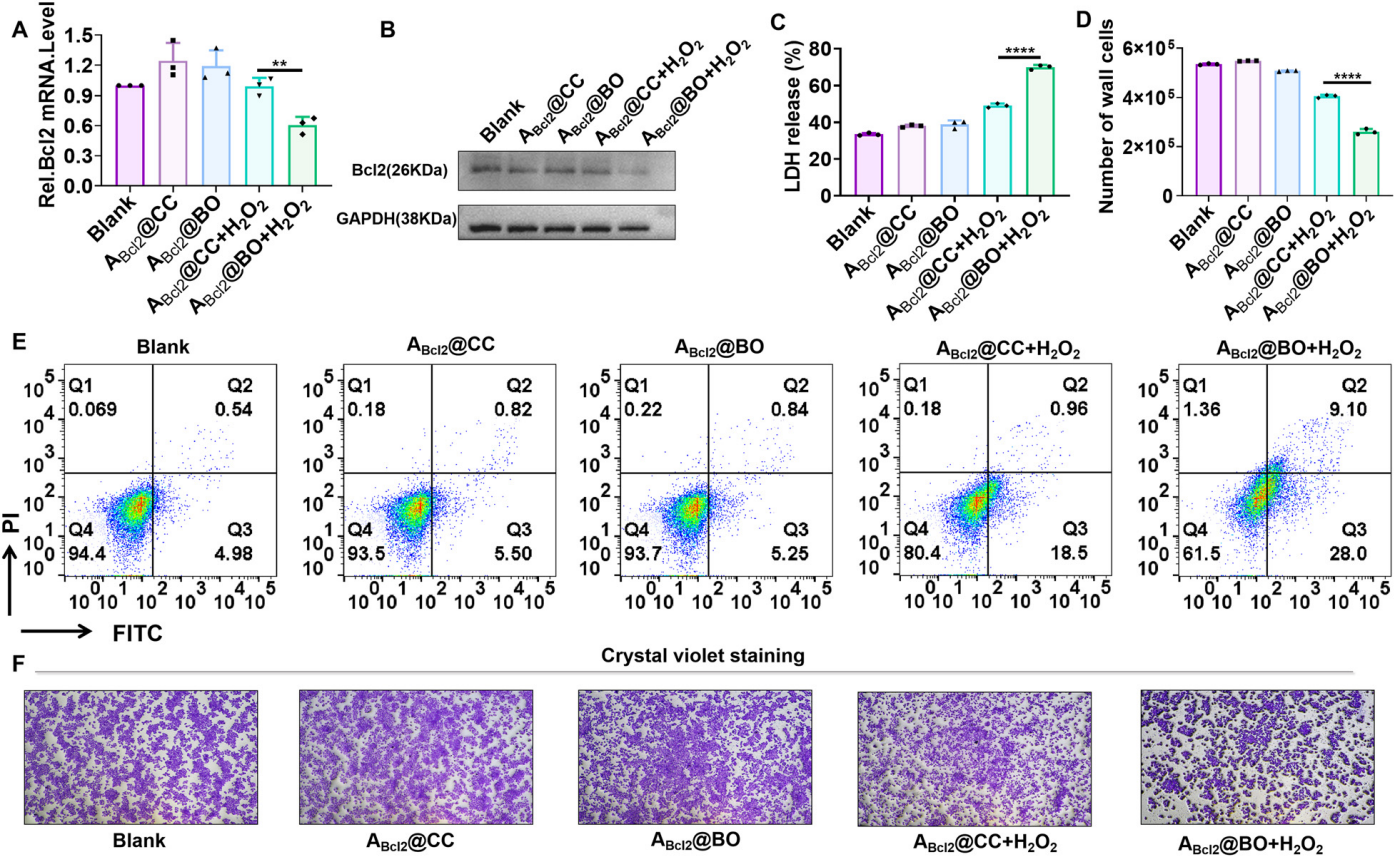
H_2_O_2_-triggered activation of A_Bcl_2__@BO for controllable gene regulation and antitumor studies in MCF-7 cells. (A) RT-PCR analysis of Bcl_2_ mRNA expression under different treatments. (B) Western blot analysis of Bcl_2_ protein expression levels. Cell viability analysis by (C) the LDH release assay and (D) direct cell counting. (E) Cell apoptosis analysis using Annexin V/PI staining. (F) Cell survival evaluation by crystal violet staining. Error bars represent standard deviation from three parallel experiments. Statistical significance was calculated with a two-tailed Student's *t*-test: ****P < 0.0001.

We then assessed the therapeutic efficacy of A_Bcl_2__@BO in human breast cancer MCF-7 cells. Reverse transcription-polymerase chain reaction (RT-PCR) analysis revealed a significant reduction in Bcl_2_ mRNA expression in cells sequentially treated with A_Bcl_2__@BO and H_2_O_2_ compared to untreated cells (Fig. 3A), whereas a minimal decrease was observed in the control groups (A_Bcl_2__@BO alone, A_Bcl_2__@CC and A_Bcl_2__@CC + H_2_O_2_). Western blot analysis confirmed this result at the protein level (Fig. 3B). These results demonstrate the H_2_O_2_-triggered activation of A_Bcl_2__@BO for controlled gene silencing. We further investigated the effect of Bcl_2_ silencing on cell viability. Our data show that the sequential treatment of MCF-7 cells with A_Bcl_2__@BO and H_2_O_2_ led to a significantly increased release of lactate dehydrogenase (LDH) compared to the control groups (Fig. 3C). Consequently, a substantial reduction in the viable cell count was observed following co-treatment with A_Bcl_2__@BO and H_2_O_2_ (Fig. 3D). Moreover, the PI/Annexin V-FITC apoptosis assay demonstrated that the H_2_O_2_-activated A_Bcl_2__@BO induced a higher apoptosis rate than the control groups (Fig. 3E), which aligned with the results from the crystal violet assay (Fig. 3F). Collectively, these results demonstrate that the engineered H_2_O_2_-activated iASO enabled precise gene silencing in living cells and exhibited potential for application in cancer gene therapy.

## Conclusions

In this study, we developed a H_2_O_2_-activatable iASO by site-specifically modifying its backbone with BO caging groups. The resulting iASOs demonstrated not only enhanced metabolic stability, but also the capacity for the precise control of their functions *via* H_2_O_2_-triggered activation. Our results showed that the BO-modified iASOs remained biologically inactive in normal cells, but they effectively silenced EGFP mRNA in tumor cells with elevated H_2_O_2_ levels. Furthermore, we demonstrated that the H_2_O_2_-activated iASOs targeting the endogenous Bcl_2_ mRNA significantly induced apoptosis using a lipid nanoparticle system, thereby confirming their therapeutic potential. Therefore, our strategy provides a simple and robust platform for cell-specific gene regulation, underscoring its promise in minimizing off-target effects and advancing precision oncology.

## Author contributions

Zhen Xun and Yang Hai contributed equally to this work. Zhen Xun performed the experiments and analysed the data; Yang Hai performed the organic synthesis; Li-Juan Tang discussed and wrote the manuscript; Jian-Hui Jiang discussed the results; Zhenkun Wu designed the project and wrote the manuscript.

## Conflicts of interest

There are no conflicts to declare.

## Supplementary Material

CB-OLF-D5CB00186B-s001

## Data Availability

The data supporting this article, including DNA sequences, gel electrophoresis analysis, confocal images,and flow cytometric analysis, have been included in the supplementary information (SI). See DOI: https://doi.org/10.1039/d5cb00186b.
