# Supplementary material for: Chemically inducible antisense oligonucleotides for cell-specific gene silencing
Source: RSC Chem Biol. 2025 Oct 9;6(12):1920–6. doi: 10.1039/d5cb00186b (PMC12538225; doi:10.1039/d5cb00186b)
Supplement: CB-006-D5CB00186B-s001 [file CB-006-D5CB00186B-s001.pdf]

Supplementary Information (SI) for RSC Chemical Biology.
This journal is © The Royal Society of Chemistry 2025

Supporting Information

Chemically Inducible Antisense Oligonucleotides for Cell-Specific Gene Silencing

Zhen Xun,[#] Yang Hai,[#] Li-Juan Tang, Jian-Hui Jiang, Zhenkun Wu[*]

State Key Laboratory of Chemo and Biosensing, College of Chemistry and Chemical Engineering, Hunan University, Changsha 410082, China. E-mail: tomwu@hnu.edu.cn

Table of Contents

Materials and methods……………………………………………..…..S1

Materials…………………………………………………………...S1

Instruments………………………………………………………...S1

Methods……………………………………..……………..S2

Table 1……………………………………………………….…….…...…S8

Figures S1-S10………………………………...…………….........S9-S21

Raw image…………………………………...…………….......…....S22-S23

Materials and Methods

Materials

All chemicals used in this study were purchased from Tansoole (Shanghai, China) unless otherwise specified. 4-(Bromomethyl)phenylboronic acid and 4-(bromomethyl)phenylacetic acid (CC) was obtained from Aladdin (Shanghai, China). 30% aqueous hydrogen peroxide solution (H_2O_2) was acquired from Sigma-Aldrich (Missouri, USA). The Enhanced Cell Counting Kit-8 was purchased from APExxBIO (Houston, USA). The Annexin V-FITC Apoptosis Detection Kit, Cytotoxicity Assay Kit, and Hydrogen Peroxide Content Assay Kit were procured from Beyotime Biotechnology (Shanghai, China). Lipofectamine 3000 was obtained from Thermo Fisher (Massachusetts, USA). Opti-MEM was purchased from Invitrogen (Massachusetts, USA). Dulbecco's Modified Eagle Medium (DMEM), phosphate-buffered saline (PBS), fetal bovine serum (FBS), 100 IU/mL penicillin-streptomycin, and 0.25% trypsin were acquired from Thermo Fisher Scientific (Massachusetts, USA). Evo M-MLV Reverse Transcription Kit with gDNA Removal Reagent for qPCR (AG11705) and SYBR Green Pro Taq HS Premixed qPCR Kit (AG11701) (Changsha,China). Rabbit monoclonal anti-Bcl2 and rabbit polyclonal anti-GAPDH antibodies were obtained from Immunoway (Suzhou, China). MCF-7, Ncl-H460 and HEK293T cell lines were procured from the Cell Bank of the Chinese Academy of Sciences Committee on Type Culture Collection (Shanghai, China). Ultrapure water with resistivity >18.2 MΩ was prepared using a Millipore Milli-Q water purification system (Billerica, Massachusetts, USA). Oligonucleotides were synthesized by Sangon Biotech Co., Ltd (Shanghai, China).

Instruments

HPLC (Agilent 1260); Freeze dryer (SCIENTZ-10N/A); PCR Amplifier (960-T, Hangzhou Jingge Science Instrument Co.); Chemiluminescence imager and Gel imager (Sage, Beijing).

Methods

General procedure for construction of inducible ASOs

A_{GFP-8}@BO, A_{Bcl2}@BO, and A_{Bcl2}@CC were synthesized via high-efficiency Br-PS chemistry by conjugating PS-modified A_{GFP-8} and A_{Bcl2} with BO or CC .Briefly, BO or CC was dissolved in dimethyl sulfoxide (DMSO) at a concentration of 25 mM. Meanwhile, A_{GFP-8}@PS and A_{Bcl2}@PS were dissolved in 1× phosphate buffer (PB; 50 mM phosphate, pH 7.0) at a concentration of 100 μM. The two solutions were mixed at a 1:1 molar ratio and magnetically stirred at 37℃ for 48 h. The resulting reaction mixture was collected and lyophilized to remove organic solvents. The crude product was then redissolved in ultrapure water and purified using an Amicon Ultra-3K centrifugal filter (Millipore) with repeated washing to remove excess BO or CC.

PAGE gel electrophoresis assay

The interaction assays were performed by incubating the following complexes at equimolar concentrations (100 nM each): A_{GFP-8}@4BO, A_{Bcl2}@BO and A_{Bcl2}@CC with their respective substrate strands (mGFP or mBcl2, 100 nM) in 1× Tris-NaCl buffer (10 mM Tris-HCl, 140 mM NaCl, pH 7.4). To investigate the effect of oxidative stress, parallel reactions were conducted in the presence or absence of 300 μM H_2O_2. All mixtures were incubated at 37℃ for 15 min to allow sufficient binding. Following incubation, the samples were analyzed by denaturing 15% SDS-polyacrylamide gel electrophoresis (SDS-PAGE). Electrophoresis was performed at a constant voltage of 130 V using TAE running buffer (40 mM Tris-acetate, 1 mM EDTA). Post-electrophoresis, the gels were stained with the fluorescent nucleic acid dye GelRed (10 min, protected from light) and visualized using a Tanon 4200SF gel imaging system under UV excitation. To ensure reproducibility, three independent biological replicates were performed for each experimental condition.

Melting curve analysis

A mixture was prepared by combining 1 μl of diluted SYBR Green I, 4 μl of

phosphate buffer (final concentration: 5 mmol/L), and 6 μl of Tris buffer (final concentration: 60 mmol/L). An appropriate volume of DNA solution was added to achieve a final DNA concentration of 10 ng/μl after quantification. The total reaction volume was adjusted to 40 μl using ultrapure water. The prepared solution was then loaded into a real-time PCR instrument, and the following program was executed: 30 °C for 30 seconds, followed by 65 cycles with a temperature increment of 1 °C per cycle. The melting curve of the DNA fragment was recorded, and the derivative melting curve was analyzed. The Tm value was determined as the temperature corresponding to the peak of the derivative melting curve.

Cytotoxicity of H_2O_2

HEK293T cells were seeded in 96-well plates at a density of 10,000 cells per well and allowed to adhere for 24 hours under standard culture conditions. Following incubation, cells were exposed to varying concentrations of H_2O_2 (0, 20, 50, 100, 200, 300 μM) prepared in sterile ultrapure water for a treatment duration of 3 hours. Cells were gently washed with 1× PBS to remove residual H_2O_2. Cellular viability was subsequently determined using the Enhanced Cell Counting Kit-8 (CCK-8) assay according to the manufacturer's protocol. The absorbance of formazan product was quantified at 450 nm using an ELx800™ microplate reader. All experiments were performed with appropriate controls and repeated in at least three independent biological replicates to ensure statistical reliability.

H_2O_2 activation of A_{GFP-8}@BO and A_{Bcl2}@BO in live cells

HEK293T cells were plated in 21 mm culture dishes containing 1 mL of DMEM growth medium supplemented with 10% FBS and 1% penicillin/streptomycin, followed by 24-hour incubation at 37℃ under 5% CO_2. Cells were then transfected with EGFP plasmid (1 μg/mL) and A_{GFP-8}@BO (300 nM) using the commercial transfection reagent Lipofectamine 3000, following the manufacturer's protocol. After 6 hours of transfection, cells were washed with 1× PBS and subsequently treated with 200 μM

H_2O_2 for 3 hours to induce oxidative stress. Following H_2O_2 treatment, cells were again washed with 1× PBS and maintained in fresh complete medium for 16 hours prior to further analysis. MCF-7 cells were cultured under identical initial conditions in 21 mm dishes with 1 mL of supplemented DMEM medium. These cells were transfected with A_{Bcl2}@BO and A_{Bcl2}@CC (1 μM each) using Lipofectamine 3000. Following 6 hours of transfection, cells were washed with 1× PBS and cultured for 6 hours in medium containing either 300 μM H_2O_2 or untreated control medium. After oxidative stress exposure, cells were washed with 1× PBS and allowed to recover in fresh complete medium for 36 hours.

Confocal imaging assay of $A_{GFP\text{-}8}$@BO in live cells

HEK293T cells were seeded in 21 mm culture dishes containing 1 mL of DMEM supplemented with 10% FBS and 1% penicillin/streptomycin. The cells were maintained at 37 °C in a humidified 5% CO_2 atmosphere for 24 hours until reaching 70-80% confluency. Cells were transfected with EGFP expression plasmid (1 μg/mL) and $A_{GFP\text{-}8}$@BO oligonucleotide complex (300 nM) using Lipofectamine 3000 transfection reagent, following the manufacturer's recommended protocol. The transfection mixture was incubated with cells for 6 hours under standard culture conditions. Next, cells were washed twice with 1× PBS to remove residual transfection complexes, then exposed to 200 μM H_2O_2 prepared in serum-free DMEM for 3 hours to induce oxidative stress. After H_2O_2 treatment, cells were thoroughly washed with 1× PBS and subsequently cultured in fresh complete growth medium for 16 hours to allow cellular recovery and transgene expression. EGFP expression was quantitatively assessed using fluorescence confocal microscopy.

Flow cytometry assay of $A_{GFP\text{-}8}$@BO in live cells

Following confocal imaging analysis, the cells were first washed twice with 1× PBS to remove residual culture medium components. Cell detachment was achieved through enzymatic treatment with 0.05% trypsin-EDTA solution at 37℃for 2-3 minutes, with

the reaction terminated by adding complete growth medium containing 10% FBS. Next, cell suspension was centrifuged at 300 g for 5 minutes at room temperature to pellet the cells. After careful aspiration of the supernatant, the cell pellet was resuspended in 1× PBS and passed through a 40 μm cell strainer to ensure single-cell suspension. EGFP expression was quantitatively analyzed using a CytoFLEX flow cytometer (Beckman Coulter, USA).

H_2O_2 content detection

3000,000 cells of HEK293T cells and Ncl-H460 cells were collected, 150 μl of hydrogen peroxide detection lysate was added to each 1000,000 cells, and then placed on ice and fully lysed using an ultrasonic crusher, centrifuged at 4℃, 12000g for 5 minutes, and the supernatant was taken for subsequent determination. The next steps are to follow the hydrogen peroxide detection kit.

RT-PCR analysis of Bcl2

MCF-7 cells were seeded in 21 mm culture dishes containing 1 mL of DMEM supplemented with 10% FBS and 1% penicillin-streptomycin and incubated at 37℃ under a 5% CO_2 atmosphere for 24 hours to achieve optimal adherence and proliferation. Following the initial incubation, cells were transfected with A_{Bcl2}@BO and A_{Bcl2}@CC (1 μM each) for 6 hours using an optimized transfection protocol. After transfection, cells were washed twice with 1× PBS to remove residual transfection reagents. Subsequently, cells were exposed to either 300 μM H_2O_2 or control medium (without H_2O_2) for an additional 6 hours to evaluate the effects of oxidative stress. Following H_2O_2 treatment, cells were washed again with 1× PBS to eliminate residual oxidative agents and then cultured in fresh complete growth medium for 36 hours to allow cellular recovery. After the recovery period, total RNA was extracted using TRIzol reagent following the manufacturer's protocol. Briefly, cells were lysed directly in the culture dish with TRIzol, and RNA was isolated via phenol-chloroform extraction, followed by isopropanol precipitation. The RNA pellet was

washed with 75% ethanol, air-dried, and resuspended in RNase-free water. RNA concentration and purity were determined using a NanoDrop spectrophotometer, measuring absorbance at 260 nm (A260) and 280 nm (A280). Samples with an A260/A280 ratio between 1.8 and 2.1 were considered of high purity and suitable for downstream applications.

Western blot analysis of Bcl2

Following RT-PCR analysis, MCF-7 cells were subjected to protein extraction using RIPA lysis buffer (50 mM Tris-HCl pH 7.4, 150 mM NaCl, 1% NP-40, 0.5% sodium deoxycholate, 0.1% SDS) supplemented with complete protease inhibitor cocktail. The cell lysates were incubated on ice for 15 min to ensure complete protein solubilization, followed by centrifugation at 13,200 g for 15 min at 4℃ to remove cellular debris. The resulting supernatants were collected, and protein concentrations were quantified using a NanoDrop spectrophotometer with absorbance measurements at 280 nm. Equal amounts of protein (10 μg per lane) were resolved by SDS-PAGE under denaturing conditions. Electrophoresis was performed at constant voltage (80 V for 30 min followed by 120 V for 90 min) using 12% resolving gels. The separated proteins were then transferred to PVDF membranes (0.45 μm pore size) using wet transfer system at 100 V for 2 h in transfer buffer. Membranes were blocked with 5% (w/v) non-fat dry milk in TBST (20 mM Tris-HCl, 150 mM NaCl, 0.1% Tween-20, pH 7.6) for 2 h at room temperature with gentle agitation. Primary antibody incubation was performed overnight at 4℃ with anti-Bcl2 antibody (diluted 1:1000 in blocking buffer). After three 5-min washes with TBST, membranes were incubated with species-matched HRP-conjugated secondary antibody (1:5000 dilution) for 2 h at room temperature. Protein bands were visualized using enhanced chemiluminescence (ECL) detection kit according to the manufacturer's protocol. Chemiluminescent signals were captured using a digital imaging system with optimized exposure times. Densitometric analysis was performed using ImageJ software (NIH), with β-actin serving as loading control for normalization.

Apoptosis assays

Following RT-PCR analysis, cells were gently washed twice with ice-cold 1× PBS, and detached using 0.25% trypsin-EDTA solution (37℃, 2-3 min),Trypsinization was terminated by adding complete growth medium and collect the cells, Cell suspensions were centrifuged at 1,000 g for 5 min at 4℃ and Supernatants were carefully aspirated, Cell pellets were resuspended in 1× PBS and Washed again by centrifugation (1,000 g, 15 min, 4℃).Next, Cells were stained according to the manufacturer's protocol for [specify kit name] apoptosis detection kit and Stained cells were analyzed using CytoFLEX flow cytometer.

LDH assays

Following RT-PCR analysis, culture supernatants from both treated and control groups were collected and Cellular debris was removed by centrifugation at 12,000 g for 5 min at 4℃.Next, samples were processed according to the manufacturer's protocol for specify LDH assay kit.

Crystal violet staining

Following RT-PCR analysis, cells were gently washed twice with 1× PBS to remove residual medium components and cells were fixed with 0.1% (w/v) crystal violet solution in deionized water for 30 minutes at room temperature (25 ± 2°C).Next, cells were washed 3-5 times with 1× PBS until the wash solution became clear and stained cells were examined under an inverted optical microscope (Olympus IX73) using 10× and 20× objectives.

Cell counting experiments

The most traditional approach utilizes a hemocytometer, a specialized counting chamber with a gridded slide. A diluted cell suspension is loaded into the chamber, and

cells within predefined squares are enumerated under a microscope. The total cell count per unit volume is calculated using the formula:

$$\text{Cell concentration (cells/mL)} = \frac{\text{Number of cells counted} \times \text{Dilution factor}}{\text{Volume counted(mL)}}$$

Table1 Oligonucleotides used in this work**.**

Name	Sequence (5'→3')	Theoretical MS	Observed MS
A_{GFP-1}	GAGCTGCACGCTGCCGTC	5476.6	5475.9
A_{GFP-2}	TTGTGGCCGTTTACGTCGCC	6091.0	6090.6
A_{GFP-3}	AACTTGTGGCCGTTTACGTC	6099.0	6098.6
A_{GFP-4}	GTGCGCTCCTGGACGTAGCC	6110.0	6109.2
A_{GFP-5}	GAGCTGCACGCTGCCGTC	5476.6	5475.8
A_{GFP-6}	ATGTTGCCGTCCTCCTTGAA	6059.0	6058.2
A_{GFP-7}	AAAGAAGTCGTGCTGCTTCA	6141.0	6140.4
A_{GFP-8}	TTGCCGGTGGTGCAGATAAA	6197.0	6196.2
A_{GFP-8}@3PS	TTGCC*GGTGG*TGCAG*ATAAA	6245.3	6246.7
A_{GFP-8}@4PS	TTGC*CGGT*GGTG*CAGA*TAAA	6261.3	6262.7
A_{GFP-8}@4PS-2	TTGC*CGG*TGG*TGCAG*ATAAA	6261.3	6262.4
A_{GFP-8}@4PS-3	TTG*CCG*GTG*GTG*CAGATAAA	6261.3	6262.6
A_{GFP-8}@4PS-4	TTGCC*GG*TG*GT*GCAGATAAA	6261.3	6262.8
G3139-nonsense	ATGGCGCACGCTGGGAGA	5589.6	5590.7
G3139-antisense	TCTCCCAGCGTGCGCCAT	5411.6	5412.5
A_{Bcl2}@PS	/i2OMeU//i2OMeC//i2OMeU/C*CCAGC*GTG CG*C/i2OMeC//i2OMeA//i2OMeU/	5597.5	5599.4
mGFP	CCTGAAGTTCATCTGCACCACCGGCAAGC TGCCCGTG	11271.4	11271.3
mBcl2	CTGGGAAGGATGGCGCACGCTGGGAGAA CAGGGTAC	11265.2	11266.3
Bcl2-F	GACGACTTCTCCCGCCGCTAC	6303.2	6304.2
Bcl2-R	TCCCCCAGTTCACCCCGTCC	5910.0	5910.9
GAPDH-F	GTTCCTACCCCCAATGTGTCC	6293.2	6294.4
GAPDH-R	TAGCCCAAGATGCCCTTCAGT	6366.2	6367.5

*: phosphorothioate (PS) modified DNA backbones.
/i2OMeU/, /i2OMeC/, /i2OMeA/: 2′-O-methoxy-modified nucleotides

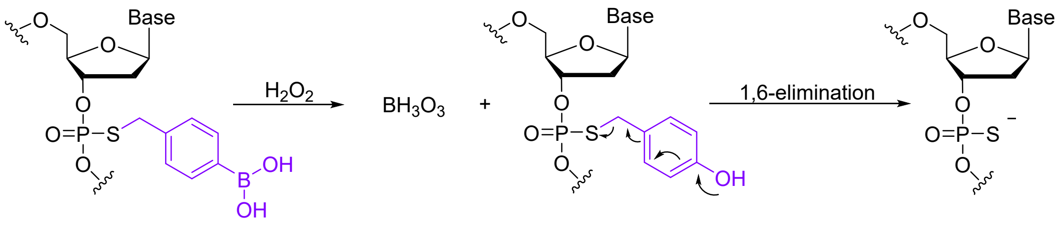

Fig. S1 Mechanism of H_2O_2-triggered removal of BO caging groups.

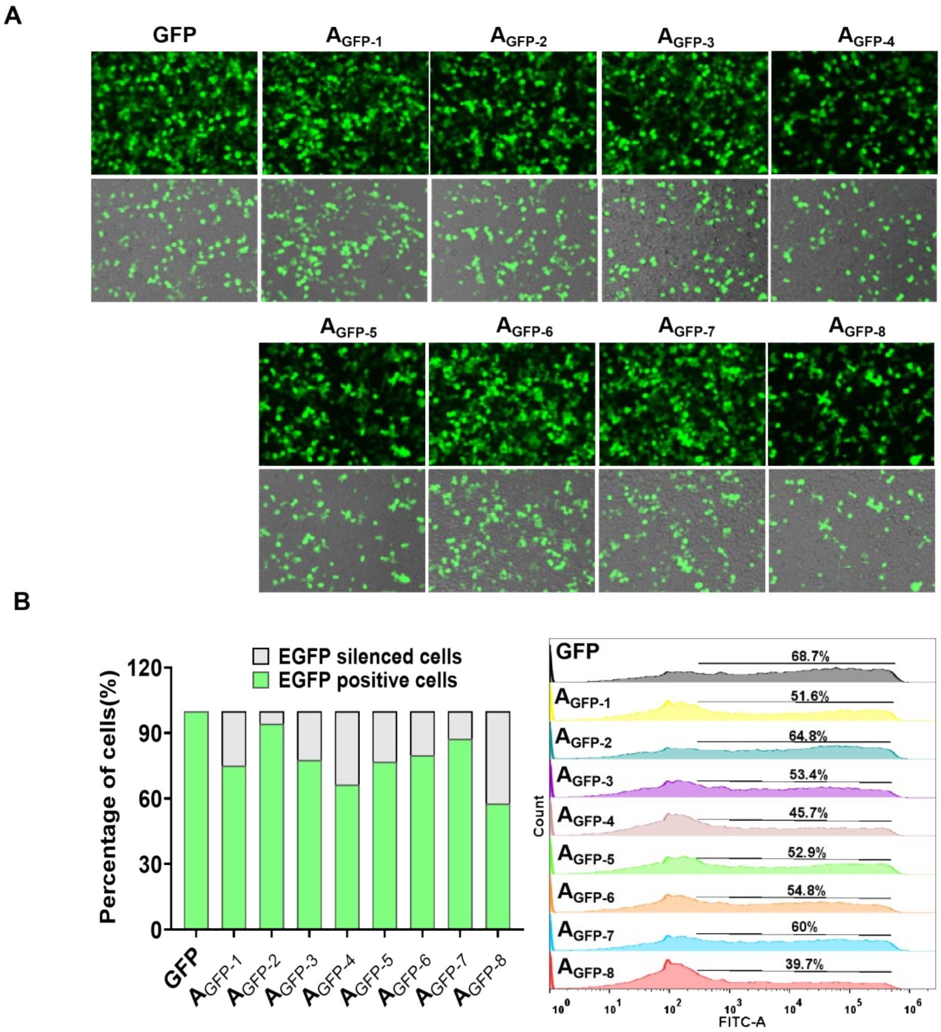

Fig. S2 Evaluation of the effects of eight A_{GFP} oligonucleotides on GFP expression in HEK293T cells using Confocal imaging (A) and flow cytometry (B).

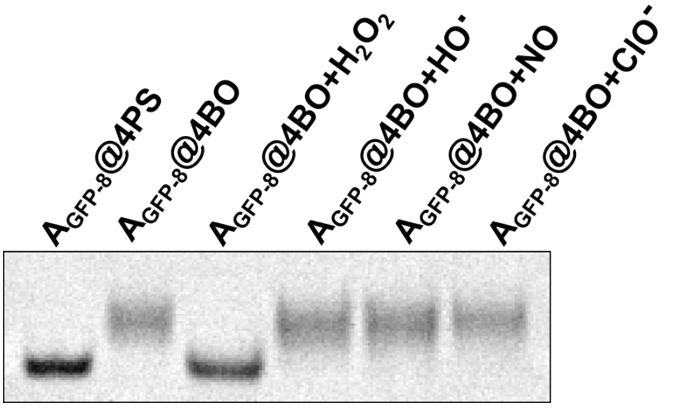

Fig. S3 Gel imaging analysis evaluating the responsive specificity of A_{GFP-8}@4BO. The concentration of H_2O_2, HO•, NO, and ClO^- were 300 μM each (HO• were generated by the reaction of $(NH_4)_2Fe(SO_4)_2$, EDTA with H_2O_2; NO was generated from PROLI NONOate.).

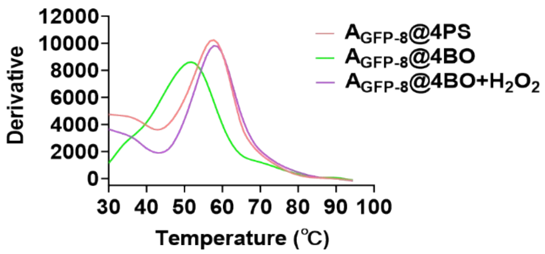

Fig. S4 DNA melting analysis (first derivative of the melting curves) of A_{GFP-8}@4PS, A_{GFP-8}@4BO and H_2O_2-activated A_{GFP-8}@4BO.

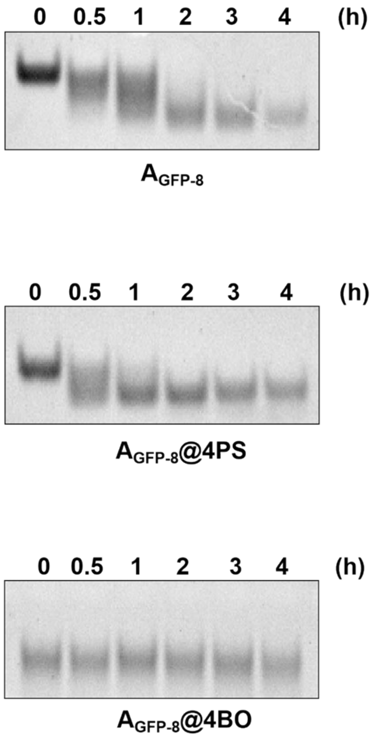

Fig. S5 Gel imaging analysis evaluating the serum stability of A_{GFP-8} (top), A_{GFP-8}@4PS (middle), and A_{GFP-8}@4BO (bottom).

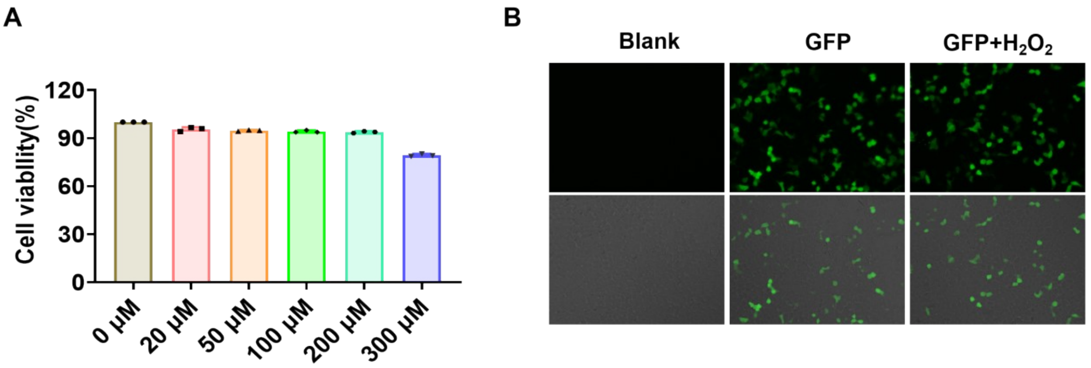

Fig. S6 (A) Tolerance of HEK293T cells to varying concentrations of H_2O_2. (B) The effect of H_2O_2 on GFP expression, as analyzed by Confocal imaging.

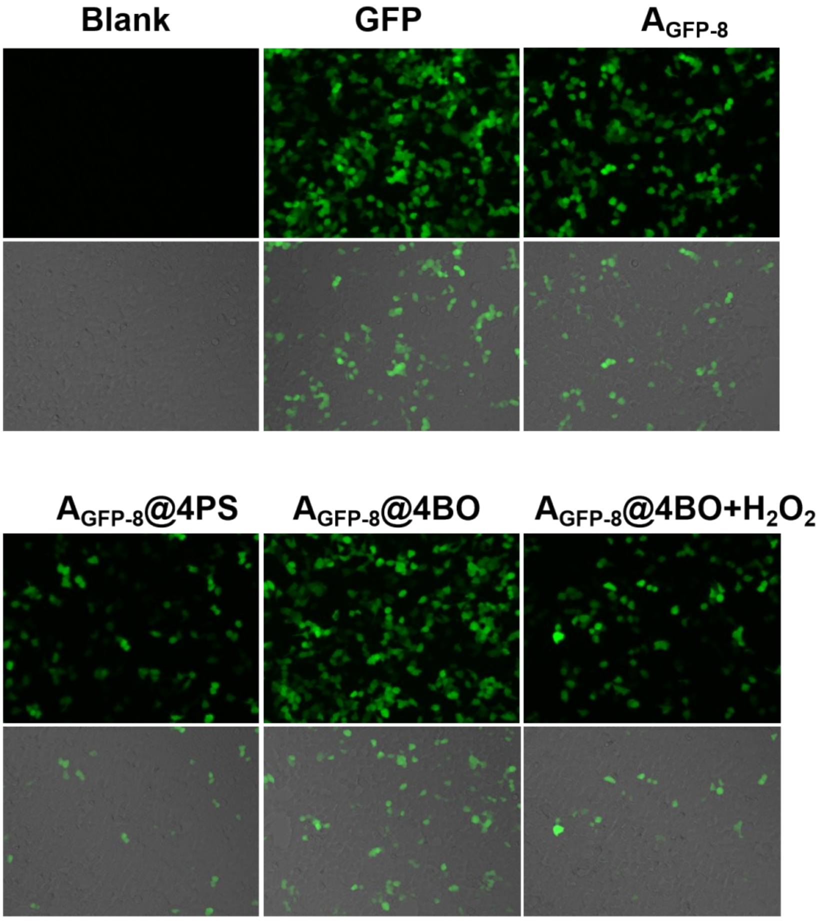

Fig. S7 Evaluation of the effects of A_{GFP-8}, A_{GFP-8}@4PS and A_{GFP-8}@4BO on GFP expression in HEK293T cells.

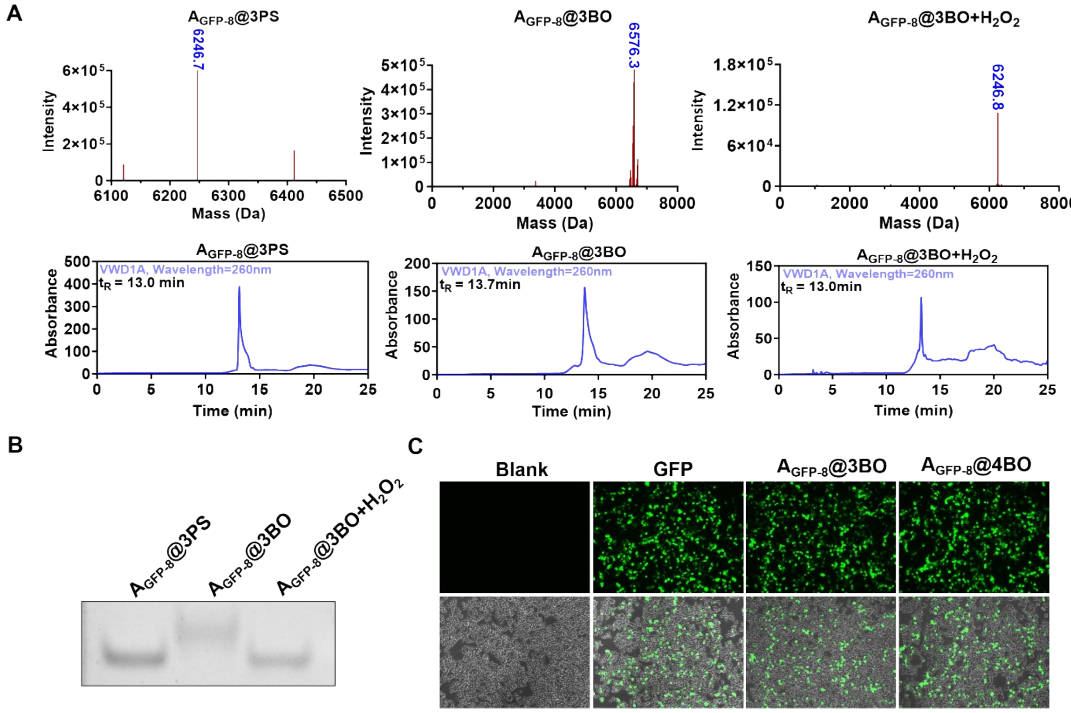

Fig. S8 (A) Characterization of A_{GFP-8}@3BO by HPLC (bottom panel) and MS (top panel). (B) Gel imaging analysis evaluating the H_2O_2 responsiveness of A_{GFP-8}@3BO (300 μM H_2O_2, 100 min). (C) The effects of A_{GFP-8}@3BO and A_{GFP-8}@4BO on GFP expression.

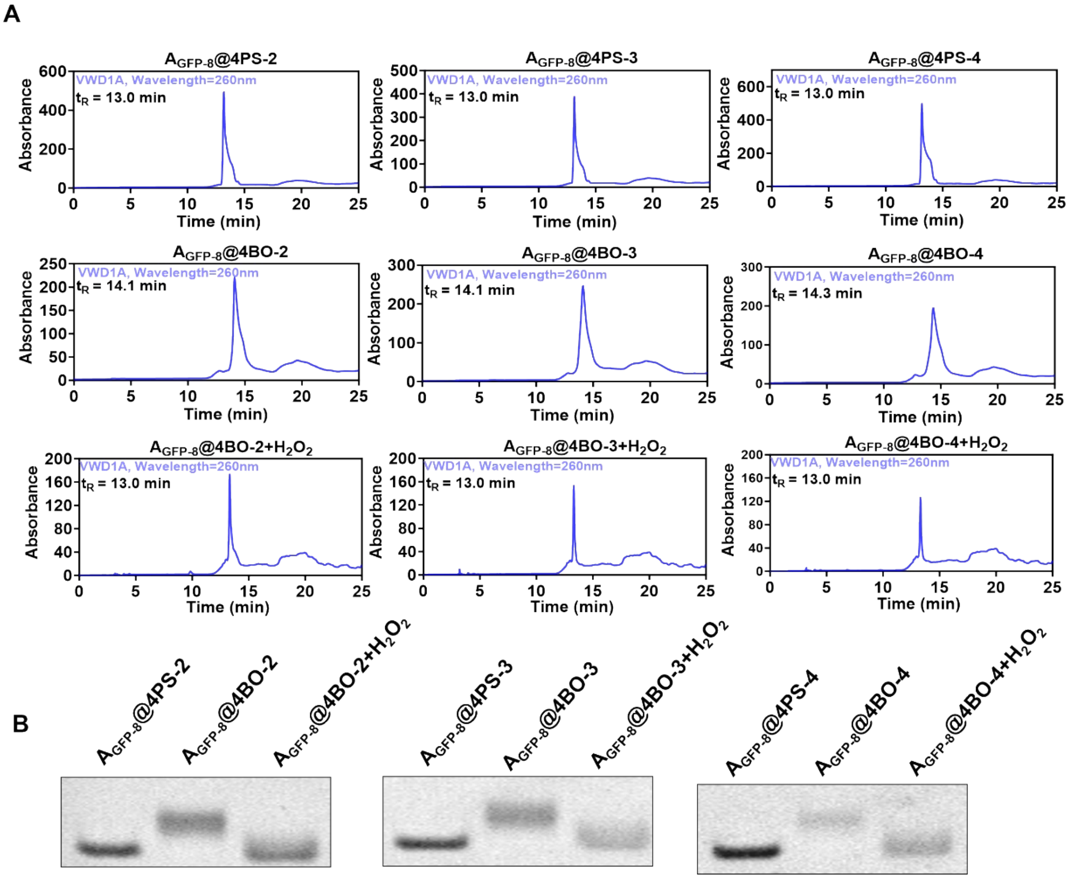

Fig. S9 (A) Characterization of A_{GFP-8}@4BO variants by HPLC. (B) Gel imaging analysis evaluating the H_2O_2 responsiveness of A_{GFP-8}@4BO variants (300 μM H_2O_2, 100 min).

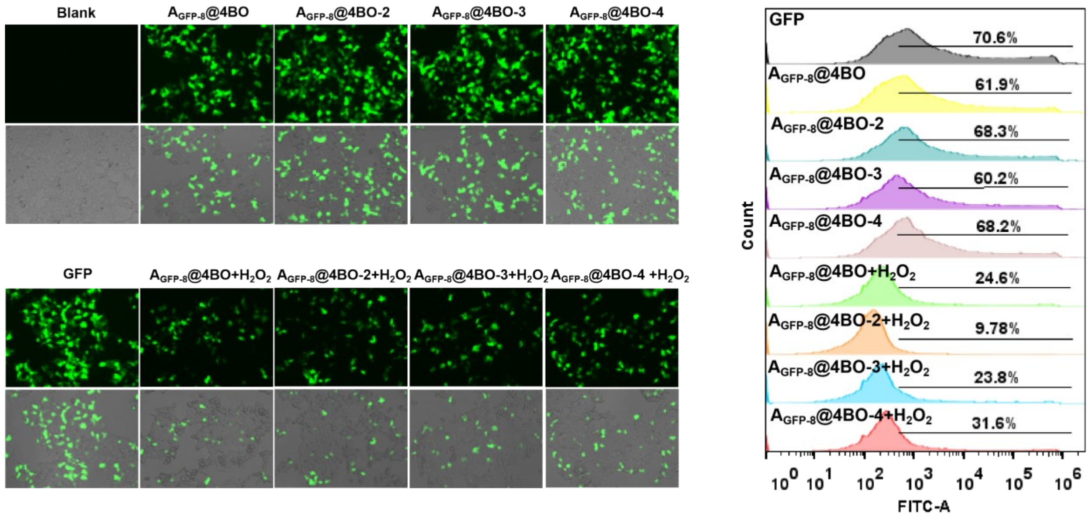

Fig. S10 The effects of four $A_{GFP\text{-}8}$@4BO variants on GFP expression levels simulated with or without H_2O_2, as characterized by confocal imaging (left) and flow cytometric analysis (right).

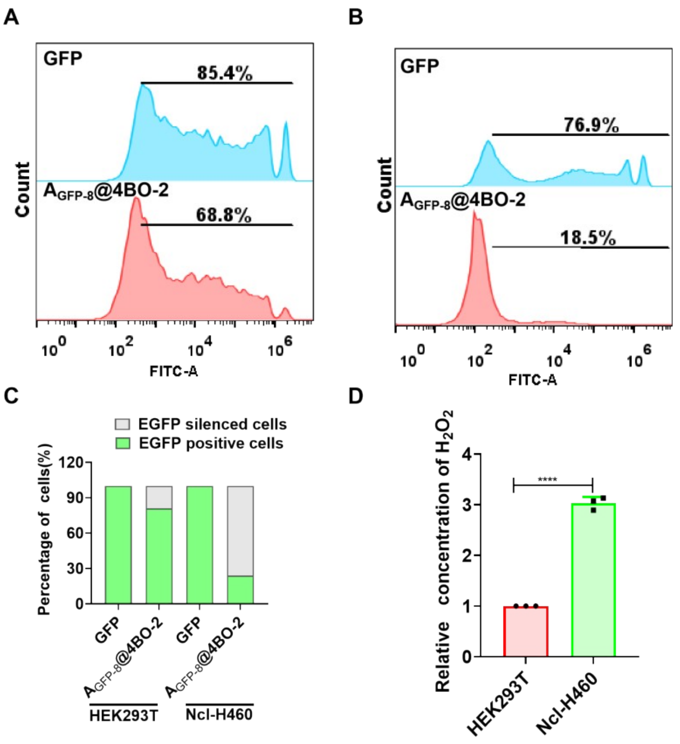

Fig. S11 (A-C) Comparison of the effects of A_{GFP-8}@4BO-2 on GFP expression in HEK293T cells (A) versus Ncl-H460 cells (B). (D) The intracellular H_2O_2 levels within HEK293T and Ncl-H460 cell lines.

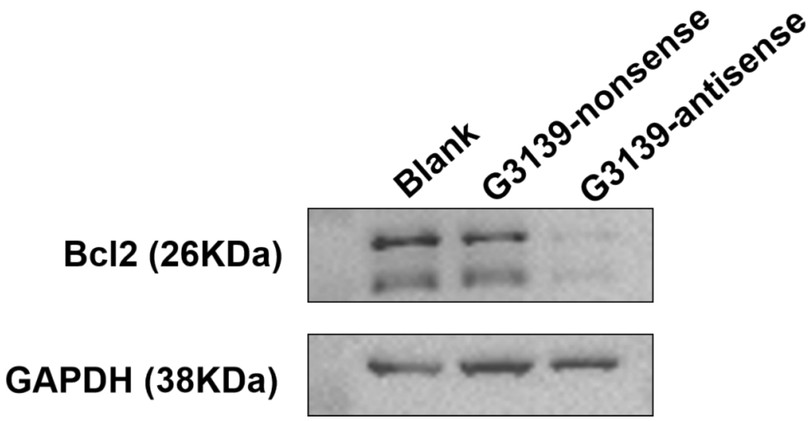

Fig. S12 The silencing effect of G3139 on Bcl-2 expression (G3139-nonsense is a Bcl2 non-targeting strand, G3139-antisense is a Bcl2-targeting strand).

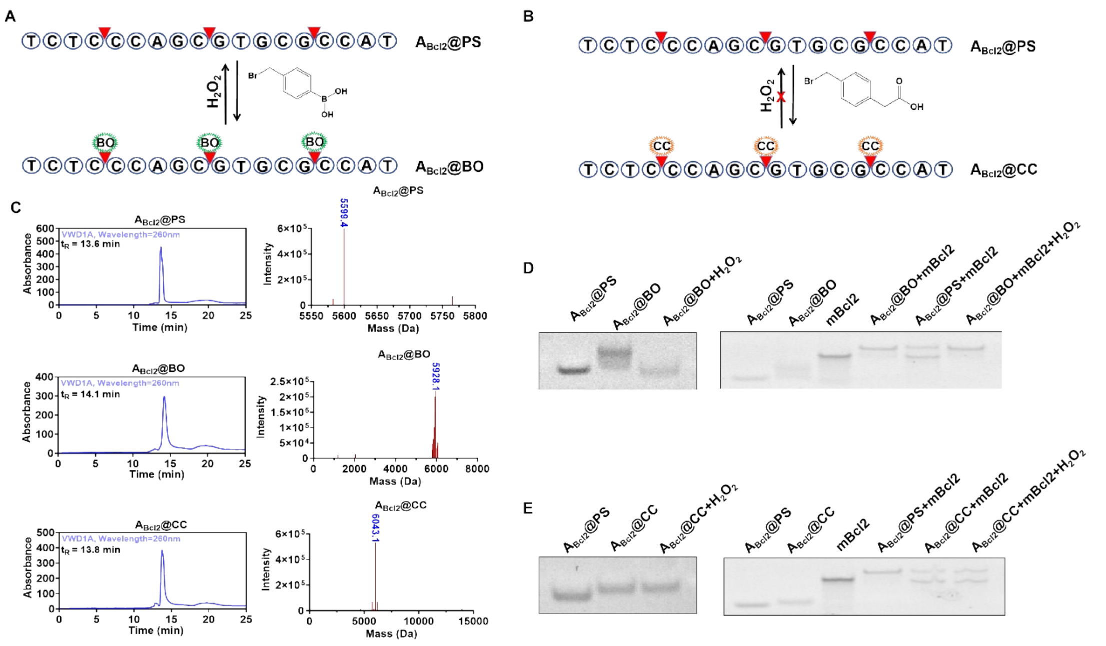

Fig. S13 (A) Schematic illustration of the synthesis of A_{Bcl2}@BO and H_2O_2-triggered activation. (B) Schematic illustration of the synthesis of the negative control of A_{Bcl2}@CC. (C) Characterization of A_{Bcl2}@BO and A_{Bcl2}@CC HPLC (left column) and MS (right column). (D) Gel imaging analysis evaluating the H_2O_2 responsiveness of A_{Bcl2}@BO and the ability of A_{Bcl2}@BO for controlled mRNA hybridization. (E) Gel imaging analysis evaluating the H_2O_2 responsiveness as well as the mRNA hybridization capacity of the negative control of A_{Bcl2}@CC.

Uncropped/full-size gel/blot for Figures in the main text and SI

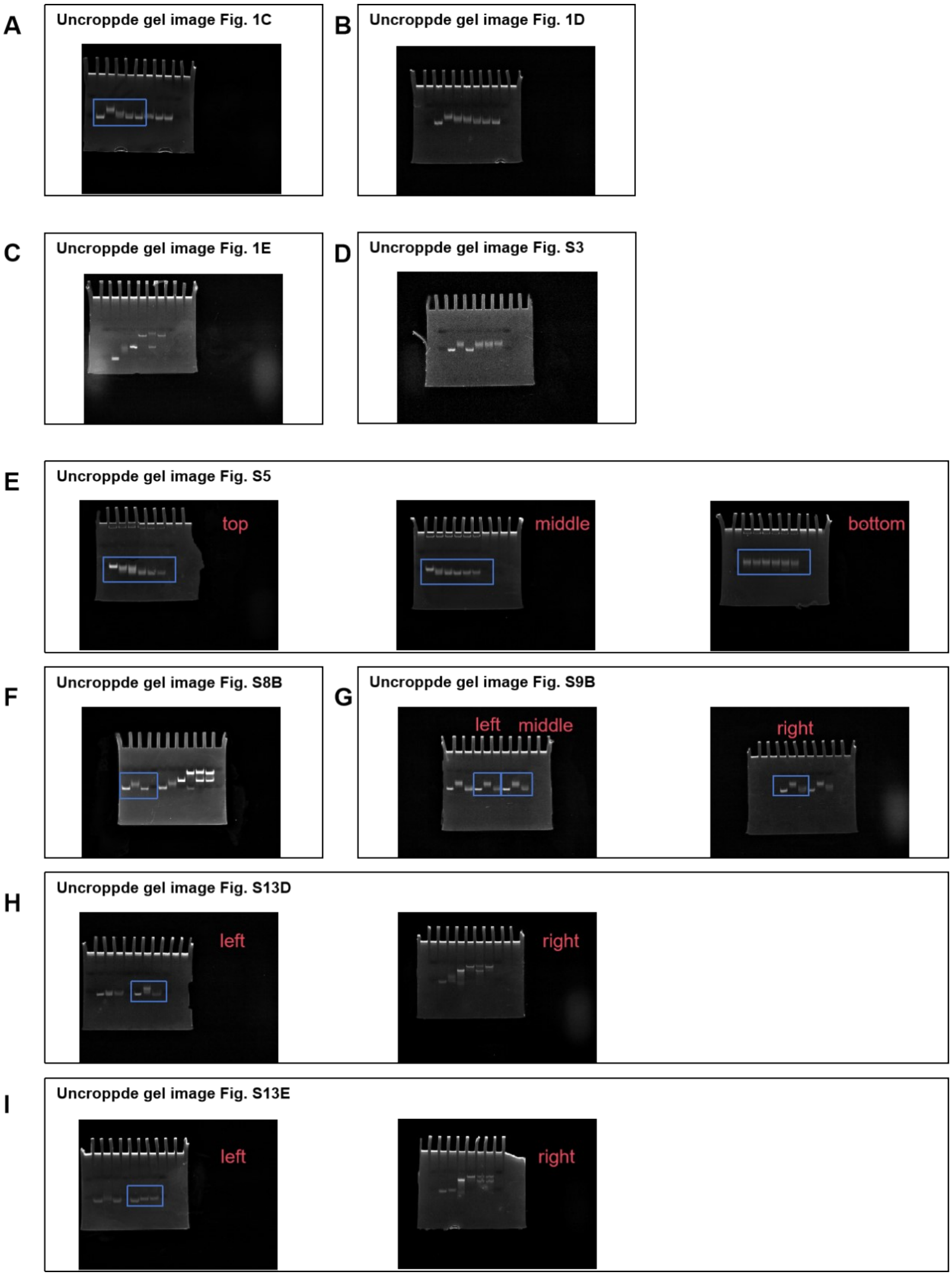

Fig. S14 Uncropped gel images for (A) Fig. 1C, (B) Fig. 1D, (C) Fig. 1E, (D) Fig. S3, (E) Fig. S5, (F) Fig. S8B, (G) Fig. S9B, (H) Fig. S13D and (I) Fig. S13E.

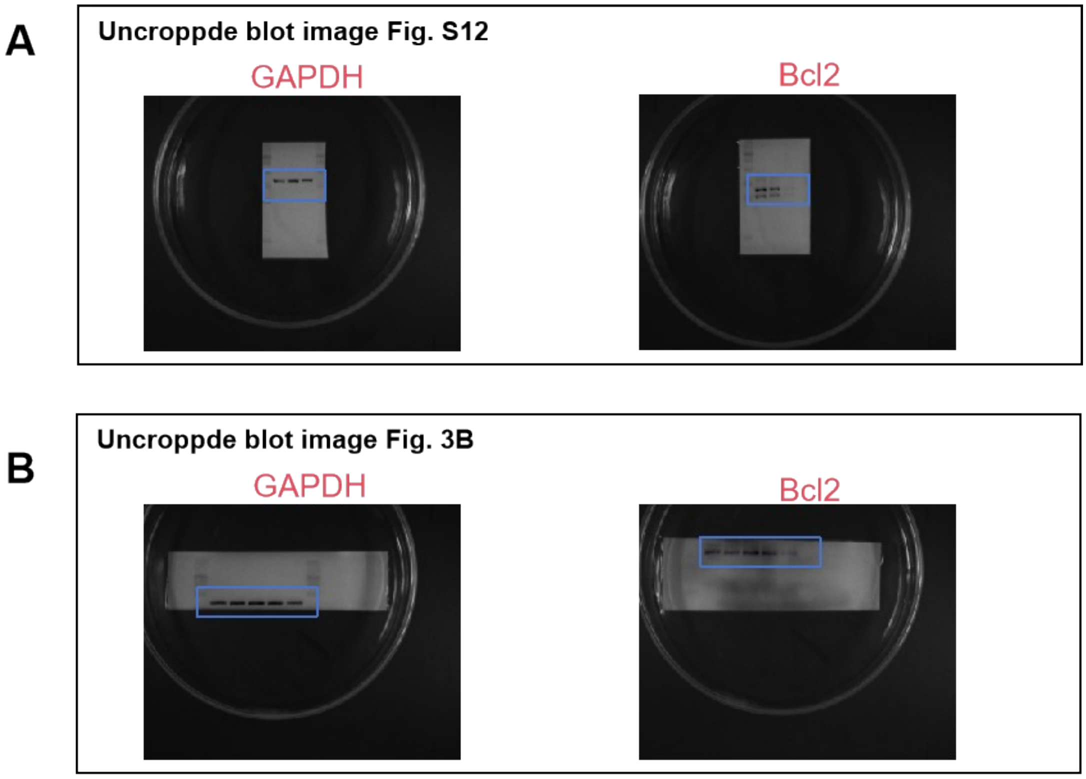

Fig. S15 Uncropped blot images for (A) Fig. S12, (B) Fig. 3B.
